# Effectiveness and Safety of Direct-Acting Antivirals in the Treatment of Chronic Hepatitis C: A Real-life Study in Northeastern Brazil

**DOI:** 10.1590/0037-8682-0192-2024

**Published:** 2024-11-25

**Authors:** Elodie Bomfim Hyppolito, Alberto Novaes Ramos, Larissa Peixoto Teixeira, Arthur Machado Bezerra, Lucas Arruda Mendes, Taynara Lais Silva, José Milton de Castro Lima, Érico Antonio Gomes de Arruda, Eder Janes Guerra, Maria Macedo Saraiva Tavares, Carlos Eduardo Pereira Lima, Ticiana Mota Esmeraldo, Francisco Sérgio Rangel de Paula Pessoa, Alessandra Maria Montalverne Pierre, Karla Brandão Pereira, Antônio Haroldo Araújo, Lívia Melo Carone Linhares, Anderson Fuentes Ferreira, Roberto da Justa Pires

**Affiliations:** 1 Universidade Federal do Ceará, Programa de Pós-Graduação em Saúde Pública, Fortaleza, CE, Brasil.; 2 Universidade Federal do Ceará, Departamento de Cirurgia Digestiva, Unidade de Transplante de Fígado, Fortaleza, CE, Brasil.; 3 Hospital São José de Doenças Infecciosas, Secretaria Estadual de Saúde do Ceará, Fortaleza, CE, Brasil.; 4 Universidade de Fortaleza, Fortaleza, CE, Brasil.; 5 Hospital Geral de Fortaleza, Secretaria Estadual de Saúde do Ceará, Fortaleza, CE, Brasil.

**Keywords:** Chronic Hepatitis C, Antiviral Drugs, Sustained Virologic Response, Epidemiology

## Abstract

**Background:**

This study aimed to evaluate the effectiveness and safety of direct-acting antivirals (DAAs) for hepatitis C treatment by measuring sustained virologic response (SVR) and serious adverse events to help design effective interventions for reducing disease prevalence.

**Methods:**

This was a retrospective, observational, real-life study of patients with chronic hepatitis C receiving DAA treatment in the state of Ceará, Brazil. Data were collected in REDCap and analyzed using R® software by the Student's t, chi-square, and Fisher’s exact tests, with a significance level of 5%.

**Results:**

In this study, 1075 patients who were diagnosed with hepatitis C infection between October 2015 and October 2023 were included. The mean age of the participants was 56.6 ± 11 years and 60.2% were men. The sample included 51 HIV-infected patients (6.6%), 166 (15,4%) liver transplant recipients, 34 (3,1%) kidney transplant recipients, and 446 patients with cirrhosis (41.4%). The overall SVR rate was 96.4%. The sofosbuvir/daclatasvir/ribavirin regimen used in 354 (32.9%) patients achieved an SVR of 96%. The cure rate was 96.5%, with a lower SVR in patients with cirrhosis (93.4%) than in those with less severe fibrosis (97.9%) *(p*=0.0015). Serious adverse events associated with ribavirin use occurred in 3.5% of patients.

**Conclusions:**

DAA treatment for hepatitis C achieved SVR in real life in all patient profiles, including transplant recipients, HIV carriers, and patients with cirrhosis. Although these drugs are safe, a few decompensated patients with cirrhosis died during treatment.

## INTRODUCTION

Hepatitis C virus (HCV) infection is a silent disease that leads to chronic liver inflammation and potentially cirrhosis and hepatocellular carcinoma (HCC) in 20-30% of cases. According to a recent global report on hepatitis, only 20% of individuals with HCV infection are aware of their status and only 8% of those diagnosed with the disease are currently undergoing antiviral therapy^1^.

In Brazil, the prevalence of HCV infection among individuals aged 15-69 years is estimated to be between 0.4% and 0.7%, corresponding to approximately 700,000 individuals requiring treatment. HCV is the most commonly reported viral hepatitis infection in the country and is the leading cause of indication for liver transplantation associated with HCC, although treatment uptake is generally low^2^. In 2016, the World Health Organization³ proposed eliminating hepatitis C as a public health threat by 2030. However, achieving the goal of an 80% reduction in new chronic infections and a 65% decrease in mortality from the 2015 levels would require ≥90% of individuals with HCV to be diagnosed and ≥80% to be treated^3^.

In developed countries, the burden of HCV infection, particularly new infections and reinfections, is largely borne by individuals who inject drugs. Despite the widespread use of safe and sensitive diagnostic tools for hepatitis C and direct-acting antivirals (DAAs), elimination of HCV infection remains a challenge in developed countries implementing WHO strategies. Urgent revisions of uptake and treatment strategies are needed to address all risk groups within the healthcare system and eradicate this public health problem^4^.

Hepatitis C treatment has evolved significantly over the past two decades. Interferon monotherapy was used in the 1980s, with cure rates <10%; however, when combined with ribavirin (RBV), the sustained virological response (SVR) improved to 30-50%^5^. Protease inhibitors, when used in combination with interferon and RBV, slightly enhanced SVR rates; however, this also led to increased morbidity and mortality^6^.

In 2015, the Ministry of Health adopted DAAs for hepatitis C treatment following the Clinical Protocol and Therapeutic Guidelines for Hepatitis and Co-infections (2015). Initially, treatment involved a combination of sofosbuvir, simeprevir, and daclatasvir, with the subsequent addition of other DAAs, all provided free of charge by the Unified Health System (from the Portuguese acronym SUS - *Sistema Único de Saúde*). First, only patients with cirrhosis were eligible for treatment; however, with the progressive expansion of access, patients with hepatitis C with and without fibrosis were made eligible in 2018. The treatment initially consisted of a combination of sofosbuvir, daclatasvir, simeprevir, RBV, and pegylated interferon; however, over time, the so-called 3D combination has been introduced (ombitasvir/veruprevir/ritonavir/dasabuvir, sofosbuvir/velpatasvir, grazoprevir/elbasvir, and glecaprevir/pibrentasvir). This study aimed to evaluate the epidemiology, adverse events, and SVR of chronic hepatitis C treatment in a real-life setting. Additionally, we assessed the impact of the place of residence on treatment outcomes to identify difficulties in accessing diagnosis and treatment.

### ● Why was this study done?

This was a real-life retrospective cohort study evaluating the epidemiology, origin, effectiveness, and serious adverse events of patients with hepatitis C who were treated at viral hepatitis referral services in the state of Ceará, Brazil. 

### ● What did the researchers do?

The research team evaluated the effectiveness, accessibility, and serious adverse events related to medication in specialized health services in Ceará over a 7-year period to better understand which hepatitis C elimination strategies should be adopted in future. This study aimed to estimate the number of patients that have already been treated and the number of patients that need to be treated to eliminate hepatitis C in Ceará.

### ● What did the researchers find?

The results of this real-life study confirm the efficacy and safety of using DAA for hepatitis C treatment. The combination of DAA with RBV did not improve efficacy and was associated with serious adverse events.

Most treated patients lived in the capital of Ceará State. More than 90% of the patients were cured of hepatitis C. Nine patients died during the treatment.

### ● What do the results mean?

This study documents that, currently, the greatest difficulty in eliminating hepatitis C is diagnosis and access to treatment, especially for patients living far from specialized viral hepatitis treatment centers. Treatment is generally safe and requires caution in patients with decompensated cirrhosis, in whom treatment should be postponed until after liver transplantation. We hope that this study reinforces the need for the decentralization of public health policies designed to eliminate hepatitis C.

### ● Key concepts and learning points

- This was a real-life study involving a large number of patients with hepatitis C from three outpatient services in a northeastern Brazilian state capital documenting the effectiveness of hepatitis C treatment with DAAs.
- Patients with cirrhosis, HIV carriers, liver and renal transplant recipients, and patients with chronic renal failure were well-represented in the sample.
- The low proportion of patients with hepatitis C from the hinterlands in our sample underscores the importance of decentralizing specialized care to eliminate hepatitis C in the state of Ceará.

## METHODS

### ● Study design

This retrospective observational study was conducted in a real-life setting and focused on patients with chronic hepatitis C treated with DAAs in Brazilian public hospitals between October 2015 and December 2023. This study included nearly all the patients undergoing hepatitis C treatment in the state of Ceará. Data from all patients treated in the state of Ceará were accessed directly from the Ceará State Health Department.

### ● Study population

According to the Health Department of the state of Ceará, 1734 treatments with DAAs for hepatitis C were distributed during the study period. A total of 1522/1734 (87.8%) patients were included in this study between October 2015 and October 2023. Notably, 447 patients were excluded from the effectiveness analysis due to a lack of outcome information, leaving a final sample of 1075 patients. The initial sample comprised 1522 patients with chronic hepatitis C infection (detected by RNA PCR) from three public hospitals in Fortaleza (São José Hospital, Walter Cantídio University Hospital, and Fortaleza General Hospital). Patients aged <18 years and those who declined to participate were excluded. In addition, 447 participants who could not be assessed for SVR (defined as HCV undetectability by RNA PCR 12 weeks after the end of treatment) were excluded, resulting in a final sample of 1075 individuals for the treatment effectiveness analysis. The sample was stratified according to place of residence (state capital *vs*. hinterland *vs*. other states). Majority of treated patients resided in the capital of Ceará state. More than 90% of the patients were cured of hepatitis C. Nine patients died during the treatment.

### ● Data collection

Data were retrieved online from the databases of the three participating hospitals. Any previous or missing data were obtained and confirmed by reviewing the medical records of each patient. The attending physician chose the treatment based on the options offered by the Brazilian public health system. History of adverse events was obtained primarily from patient charts and reports of patients who returned to refill prescriptions to hospital pharmacies.

### ● Data assessment

Demographic information, including age, sex, weight, and educational level, was collected, along with clinical and laboratory variables such as chronic diseases, pretreatment liver disease stage, presence of cirrhosis, liver biopsies, elastography, HBsAg, and anti-HIV1+2. In cases of HIV co-infection, additional data were collected on virology (RNA PCR for HCV at baseline and at least 12 weeks after treatment conclusion and HCV genotype) and treatment details (prescription date, drugs used, dosage, and use of RBV). The study parameters also encompassed the duration of treatment in weeks, any treatment prior to the current therapy, specific drugs used, adverse events during treatment, and study outcomes, including cure (if SVR was present), death, discontinuation of treatment, or loss to follow-up. Treatment failure was defined as HCV detection by RNA PCR at any time after treatment, discontinuation of treatment for any reason, or death during treatment.

### ● Statistical analysis

Study data were collected and managed using the electronic data collection and management tool REDCap^7^ hosted at the Clinical Research Unit of the University Hospital Complex of UFC. The variables are presented as mean and standard deviation, and as median, percentiles, minimum and maximum, frequency, and prevalence rate. In the analysis of the participant characteristics, the Mann-Whitney U test was used, and the data did not adhere to Gaussian distribution. Pearson’s chi-square and Fisher’s exact tests were used to investigate the association between categorical variables. A level of 5% was considered statistically significant. Statistical analyses were performed using R® statistical software^8^.

### ● Ethical considerations

The study protocol was approved by the Institutional Review Board and adhered to the tenets of the Declaration of Helsinki.

## RESULTS

The mean age of the treated patients with hepatitis C was 56.6 years (range: 27-87 years), and 60.2% (n=647) were men. The proportion of immunosuppressed male patients, n=159/216 (73,6%) (HIV-positive, n=40/51 [78%]) and those who had undergone liver transplantation, n= 119/165 [72%]), was higher in our sample than in the general population n=466/825 (56,4%) *p*<0,00001. The mean age of transplant recipients was 61 years and that of HIV-infected patients was 49 years. The mean duration of education was 9 years. No significant differences were observed in terms of age with regard to SVR. Women were cured more often than men (p=0.005). Furthermore, genotype 1 responded significantly better than genotypes 2 and 3 (p=0.007).

Genotype 1 (73.3%, n=1,047) was the most common form, particularly subtypes 1b (42.8%, n=449) and 1a (27.9%, n=293), followed by genotype 3 (24%, n=252). Information on the place of residence was available for 611 patients. The majority (n=400; 65.5%) of the 611 patients with hepatitis C treated with DAAs resided in the state capital, 167 (27.3%) lived in the hinterland, and 44 (7.2%) traveled from other states to receive a liver transplant. Nevertheless, the response to treatment was similar regardless of place of residence. When the sample was restricted to 567 patients from Ceará, 70.5% lived in the state capital and 29.5% lived in the hinterland.

Our sample included 51 HIV-infected patients (4.7%) and 165 liver transplant recipients (15.4%). In addition, when patients with liver disease were classified based on the METAVIR scale (F0-F4), F4 was predominant (n=446; 48.4%), whereas F0, F1, F2, and F3 combined accounted for 51.6%. F4 patients responded significantly worse than the other patients (p=0.0015). Patients treated for 24 weeks responded significantly better than those treated for 12 weeks (p=0.00076). Hepatitis B/C co-infection was present in 1.2% (n=13/1,075) of patients, all of whom achieved SVR without experiencing hepatitis B reactivation.

Most patients were treatment-naïve (NAIVE) (n=697, 73.9%). The remaining 245 (26.1%) patients received treatment with pegylated or conventional interferon plus RBV (n=202; 21.4%), boceprevir/telaprevir (n=33; 3.5%), or DAAs (n=10; 1%) (primarily genotype 3, patients with cirrhosis, and patients treated for only 12 weeks). Patients previously treated with DAAs responded significantly less frequently than naïve patients treated with pegylated interferons, ribavirin, boceprevir, and telaprevir (p=0.0063). The most commonly used regimen was sofosbuvir/daclatasvir/RBV (354 patients), with an SVR of 96.3%. The SVR rates were >90% for all regimens except 3D+RBV (SVR=75%; n=09/12), glecaprevir+pibrentasvir (SVR=80%; n=04/05), sofosbuvir+interferon+RBV (SVR=83.3%; n=05/06), sofosbuvir+ledispavir+RBV (SVR=89.4%; n=17/19), sofosbuvir/RBV (SVR=89.4%; n=17/19), and glecaprevir/pibrentasvir. RBV was added to DAAs in 41.7% (n=411 / 985) of cases; however, this treatment had no effect on SVR (p=0.55) (Table 1).

**TABLE 1:** Treatment effectiveness by sex, age, educational level, HCV genotype, direct-acting antiviral regimen, treatment duration, previous treatment experience, and fibrosis classification.

	N	Cure	Failure	SVR (%)	p
**Total**	**1075**	**1037**	**43**	**96.4**	0.62**
**General population**	825	791	33	95.8	
HIV	51	48	03	94.1	
Liver Tx	165	159	07	95.8	
Renal Tx	34	34	0	100	
**Sex**					0.005*
Male	647	514	33	94.9	
Female	428	418	10	97.7	
**Age (years)**					
Mean (SD)	56.6 ± 11	56.5 ± 11	59.1 ± 11		0.20***
**Education (years)**	9.03 ± 2.32	9.03 ± 2.32	9.15 ± 2.30		0.40***
**Genotype**					
1	25	25	0	100	p=0.007**
1a	293	284	9	96.9	
1b	449	438	11	97.6	
2	24	21	3	87.5	
3	252	233	19	92.5	
4	4	4	0	100	
**Treatment Regimen**					
3D	61	61	0	100	p=0.003**
SOF+VEL+RBV	1	1	0	100	
SOF+ VEL	51	50	1	98	
SOF+SIM	136	134	1	98.5	
SOF+DAC+RBV	354	341	13	96.3	
SOF+LED	78	74	4	94.8	
SOF+DAC	301	286	15	95	
SOF+LED+RBV	19	17	2	89.4	
SOF+RBV	19	17	2	89.4	
SOF+INF+RBV	6	5	1	83.3	
GLP + PBT	5	4	1	80	
3D + RBV	12	9	3	75	
**Treatment Duration**					
12 weeks	848	765	83		0.00076*
24 weeks	170	166	4		
**Previous treatment experience**					0.0063*
Naive	697	671	26	96.3	
INF+RBV	202	190	12	94	
INF+RBV+BCP or TPV	33	33	0	100	
Experienced DAAs	10	7	3	70	
**METAVIR**					0.0015*
F4	446	417	29	93.5	
Other (F0+F1+F2+F3)	477	467	10	97.9	

**SVR:** sustained virological response; **Tx:** transplant; **SOF:** sofosbuvir; **DAC:** daclatasvir; **RBV:** ribavirin; **SIM:** simeprevir; **3D:** ombitasvir+veruprevir+ritonavir+dasabuvir; **LED:** ledipasvir; **VEL:** velpatasvir; **GLP:** glecaprevir; **PBT:** pibrentasvir; **INF:** interferon; **BCP:** boceprevir; **TPV:** telaprevir; **DAAs:** direct-acting antivirals; **METAVIR:** fibrosis classification; **p-value:** indicating the probability that the observed results are due to chance, thus determining the statistical significance of the study; *:X²; **Fisher test; ***Mann-Whitney test.

Almost all patients (n=1075; 96%) were cured of their hepatitis C infections. This aligns with reported cure rates of 96% in the general population, 94.1% in HIV-infected patients, 95.8% in liver transplant recipients, and 100% in kidney transplant recipients. When patients were classified according to the Child-Pugh (Child) system, 85.1% (n=345) were categorized as Child A, 13.5% (n=55) as Child B, and only 1% (n=5) as Child C. The SVR rates in patients classified as Child A, B, and C were 94.4% (n=326/345), 89% (n=49/55), and 60% (n=3/5), respectively. The SVR was significantly higher in patients without cirrhosis (97.6%, 654/670) than in patients with cirrhosis who were categorized as Child A (p=0.016), Child B (p=0.005), or Child C (p<0.00001). When comparing the SVR among patients with cirrhosis, Child A patients had similar rates to Child B patients (p=0.2) and significantly higher rates than Child C patients (p=0.02).

Although the proportion of cured patients was higher in Child B patients than in Child C patients, the difference was not statistically significant (p=0.25). 

**FIGURE 1:**
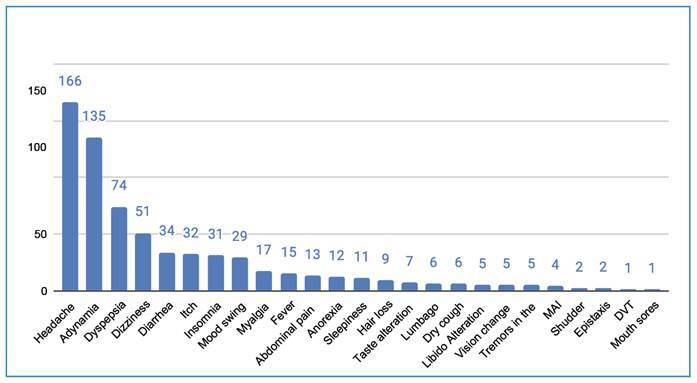
Adverse events. **MAI:** Acute Myocardial Infaction; **DVT:** Deep Vein Thrombosis.

Information on adverse events related to DAA treatment was available for 403 patients, of whom 255 (63.3%) reported no complaints or symptoms during treatment. Most patients experienced mild and transient adverse events, with headache (41%), adynamia (33%), dizziness (12.6%), and dyspepsia (8.4%) being the most common (Figure 1 **)**. Nine patients, including seven with cirrhosis and two liver transplant recipients, died. The clinical profiles and causes of death are shown in Table 2.

**TABLE 2:** Deaths during treatment with DAAs.

Sex	Age	Patient	Child	MELD	Comorbidity	Cause of death
M	63	Cirrhosis	A			HDB
M	61	Cirrhosis	A		PCT	HDB
M	35	Cirrhosis	B			Unknown case
M	65	Cirrhosis	C	15	DM	Unknown case
M	70	Cirrhosis	C	16	CI, HCC diagnosed during treatment	HCC
F		Cirrhosis	B	6		Unknown case
F		Cirrhosis	A		DM, asthma, SAH	Infection
F		LT	-	-	DM, VL	Infection
F		LT	-	-	DM, HCC	HCC relapse and infection

**DAAs:** direct-acting antivirals; **F:** female; **M:** male; **LT:** liver transplant; **A:** Child-Pugh class A; **B:** Child-Pugh class B; **C:** Child-Pugh class C; **HDB:** upper gastrointestinal bleeding; **PCT:** porphyria cutanea tarda; **DM:** diabetes mellitus; **CI:** cardiac insufficiency; **HCC:** hepatocellular carcinoma; **SAH:** systemic arterial hypertension; **VL:** visceral leishmaniasis.

The prescribed dose was recorded for 238 patients (mean: 10.7 mg/kg; range: 5-19 mg/kg). 

Severe anemia, defined as a decline in hemoglobin levels by at least 4 g, was observed in 25 of the 56 patients receiving RBV. Two patients required blood transfusion. One patient with severe anemia sustained a fall resulting in a femoral fracture. Six Child A patients with cirrhosis developed clinical decompensation during treatment. Two patients had acute myocardial infarction, one had an ischemic stroke, and one developed venous thrombosis in the lower limbs. One patient had severe diverticulitis and required colostomy, whereas one had appendicitis during treatment. Five transplant recipients showed a slight increase in liver enzymes, with biopsies revealing mild acute rejection, which was successfully managed by increasing the dose of a calcineurin inhibitor. One liver transplant recipient was lost to follow-up and developed acute renal failure, necessitating hemodialysis. In total, 4.37% (n=38/868) of the patients experienced serious adverse events during treatment. Eight patients died during treatment, and one died two weeks post-treatment (SVR was not assessed). 

A significant association (p=0.00027) was observed between RBV use and occurrence of serious adverse events. RBV was used by 67.2% (n=39/58) of the patients who experienced such events compared to 41.9% (n=376/898) of those who did not (p=0.00027).

## DISCUSSION

Since their introduction in Brazil, DAAs have proven effective in the treatment of hepatitis C. Their use resulted in SVR in approximately 96% of our patients, which aligns with the findings of another study conducted in Ceará (95%) and those from other regions of Brazil. A study involving patients from multiple Brazilian cities reported SVR rates ranging from 88% to 97%^9^ ^,^ ^10^. 

In the present study, we evaluated 12 different DAA regimens, and only five, which were used in a small number of patients (5-19 patients), achieved an SVR rate of <90%. This suggests that DAAs are highly effective against hepatitis C compared to interferon monotherapy (6% cure rate), interferon/RBV (30-60% cure rate), and protease inhibitors/interferon/RBV (54.2% cure rate)^6^. 

The efficacy of hepatitis C treatment in immunosuppressed patients (HIV carriers and liver transplant recipients) was comparable to that observed in the general population. Despite variances in demographic profiles and clinical conditions among these groups, the results indicated that DAAs could mitigate the impact of immunosuppression on HCV response^11^.

The response was lower in patients classified as F4 on the METAVIR scale than in those classified as F0-F3; however, it remained above 90%, indicating a satisfactory response. Similar findings have been published, particularly in compensated patients with cirrhosis^12^.

Most patients experienced no symptoms or only mild adverse events. Unsurprisingly, given the severity of the patients analyzed, there were cases of serious adverse events, including death. Four of the nine deaths were attributed to decompensated cirrhosis. The high prescription rate of RBV in the early years following the incorporation of DAAs in Ceará may have contributed to the occurrence of anemia.

In a study conducted in China, a significant number of treatments (19.17%) were discontinued because of digestive symptoms, such as nausea, diarrhea, and vomiting. The most frequently used DAA regimens were ledipasvir/sofosbuvir (21.86%), sofosbuvir/velpatasvir (21.77%), and sofosbuvir (13.41%)^5^. Although digestive symptoms were common in our study, the treatments were not discontinued.

A previous study reported serious adverse events, including hepatic decompensation, upper gastrointestinal bleeding, anemia, and HCC, which resulted in nine deaths and treatment discontinuation. The severity of cirrhosis has emerged as the most significant predictor of morbidity and mortality, while baseline serum albumin levels have been identified as a predictor of hepatic decompensation^13^.

A patient with cirrhosis classified as Child C developed HCC during treatment, and another patient who underwent liver transplantation for HCV and HCC experienced HCC recurrence. Although initial studies raised concerns about the potential association between HCV treatment with DAAs and an elevated risk of HCC, more recent and meticulously designed studies have refuted this hypothesis^14^.

A meta-analysis of seven trials involving RBV-containing regimens reported significant anemia during therapy (defined as hemoglobin levels of <10 g/dL). Older patients exhibited a significantly higher risk of anemia. Overall, the number of deaths reported throughout the study period was 8/1600 (0.5%) for patients aged ≥65 years and 11/4,763 (0.23%) for patients aged <65 years. Notably, four deaths recorded in our study occurred among decompensated patients with cirrhosis (Child B and C), highlighting the importance of careful patient selection^15^.

The association between adverse events and RBV has been substantiated by a meta-analysis indicating a heightened risk of anemia among older patients. Most studies evaluating RBV have consistently revealed an increased rate of adverse events. Based on these findings, and considering the overall excellent tolerability of DAAs, we believe that RBV should be avoided whenever alternative treatment options are available. With the introduction of second-generation DAA regimens such as glecaprevir/pibrentasvir, sofosbuvir/velpastavir, and sofosbuvir/velpastavir/voxilaprevir. Ribavirina is now largely avoided and rarely recommended in clinical practice guidelines^16^.

When the sample was stratified by place of residence (state capital *vs*. hinterland *vs*. other states), no significant differences in SVR rates were observed (capital=94.9% and hinterland=94.5%). In terms of demographic distribution, although two-thirds of Ceará’s population reside in rural areas, this group only represented 29% of our sample, indicating challenges in accessing the diagnosis and treatment of hepatitis C in this population. According to the 2022 Census, the state of Ceará has a population of over 8.7 million, with 2.4 million residing in the state capital, Fortaleza. This suggests that there are at least 35,000 patients with hepatitis C in Ceará, with approximately 9,700 in the capital and 35,300 in the hinterland, assuming a similar prevalence in both regions^17^. This finding reinforces the need to decentralize hepatitis C treatment in Ceará as part of a broader effort to eliminate the disease.

## CONCLUSION

The results obtained in this real-life study were consistent with those of other studies on the effectiveness of DAAs. SVR rates were considerably high across groups with different clinical and demographic characteristics. Hepatitis C treatment has been shown to be effective even among special and historically difficult-to-treat populations, such as HIV carriers, organ transplant recipients, and patients with cirrhosis. RBV addition did not increase SVR rates but led to a significant increase in serious adverse events. The limited representation of patients with hepatitis C from the hinterland underscores the need to decentralize specialized care for hepatitis C elimination in the state of Ceará.
